# An infrapatellar nerve block reduces knee pain in patients with chronic anterior knee pain after tibial nailing: a randomized, placebo-controlled trial in 34 patients

**DOI:** 10.1080/17453674.2019.1640413

**Published:** 2019-07-12

**Authors:** Pradipta Bhakta, Habib Md Reazaul Karim, Brian O’Brien, Michelle Claudio Vassallo, Mandala S Leliveld, Saskia J M Kamphuis, Michael H J Verhofstad

*Sir,—*We congratulate Leliveld and colleagues on their report (2019) of a randomized controlled trial of lidocaine as a therapeutic intervention for post-operative chronic pain following tibial nailing, and of highlighting this problematic complication. Pain that persists years after surgery is clearly difficult to treat, and the possibility that a drug as inexpensive, and readily available, as lidocaine may attenuate it is in many respects an exciting one. We wish to discuss some of the details of their study and the interpretation of their observations.

The sensory innervation of the knee is complex and variable (Bademkiran et al. 2007, Kerver et al. 2013). The surgical technique described, where, via an infrapatellar longitudinal incision, the medial aspect of the patellar ligament is accessed, may of course damage the infrapatellar genicular branch of the saphenous nerve. However, the dermatomal distribution of that nerve is largely the inferomedial aspect of the anterior knee. It would not fully account for the patterns of pain seen clinically – that is pain that covers the entire anterior knee, which is supplied by sciatic, tibial and common peroneal nerve components. Also while the authors hypothesise that nerve injury was caused by incision at the inferomedical aspect of the knee joint, they identified sensory abnormalities of the inferolateral side. These aspects of the report appear to be rather at odds with each other.

Given the complexity of the sensory innervation of the anterior knee, it is hard to view pain on kneeling as being necessarily attributable to infrapatellar nerve injury (Bademkiran et al. 2007, Kerver et al. 2013). Observations of classic findings of neuropathic pain, such as allodynia or hyperalgesia in the infrapatellar nerve distribution, were not featured in the report (Freeman et al. 2019). Direct nerve injury may be a component of course, but we suggest that the evidence presented does not describe a patient group with a homogenous clinical pain syndrome. The responses to therapy within the group vary widely, in keeping with this suggestion.

Ultimately what is described may be better seen as a diagnostic test to identify those who would benefit from longer term, or definitive, nerve blockade. The therapeutic value of local anaesthetic infiltration with a short-acting drug for pain persisting years after surgery is clearly limited, and indeed the observation that pain on kneeling is reduced by injecting the knee with that class of drug is itself unsurprising. It would presumably create numbness over some of the anterior aspect of the knee, reducing such pain. However this may be a useful method to identify those who can benefit meaningfully from longer term interventions. In that sense, rather than excluding people from a meaningful treatment prematurely, perhaps the injection might be optimally done with ultrasound guidance rather than a landmark technique. Anatomic landmarks can of course be distorted by arthritis, and in particular, years after surgery, this might be significant. Ultrasound can facilitate more accurate injection and thus a more reliable diagnostic test might ensue, identifying all who would benefit (Orduña Valls et al. 2017)

We would thank the investigators for raising this issue, and hope that such a simple test may benefit patients with long-term pain that may be treatable with more meaningful, long lasting interventions. It is, finally, worth considering if the test could be performed earlier. While they limited their study to those more than 6 months after surgery, which is often used as a definition of chronic pain, the median times reported were well over 5 years post-operatively. We would hope that this interesting study might ultimately enable more rapid identification of patients who can benefit, and thus reduce such experiences of long-term pain.

**Pradipta Bhakta, Habib Md Reazaul Karim, Brian O’Brien, and Michelle Claudio Vassallo***email:* bhaktadr@hotmail.com

*Sir,—*We thank Bhakta and colleagues for their comments on our recently published article. The infrapatellar branch of the saphenous nerve provides sensation over the anterolateral aspect of the knee and lower leg. The course of this nerve is highly variable. It crosses the patellar tendon as one or multiple branches (Kerver et al. 2013, Henry et al. 2017). The skin incision used for insertion of the tibial nail is commonly longitudinal and the nail is inserted through or just medial to the patellar tendon. Injury to infrapatellar branch at this level will cause loss of sensation at the anterolateral aspect of the knee and lower leg. Any incision (for other purposes) made more medial and cranial will cause loss of sensation of the medial aspect of the knee and calf.

Persisting anterior knee pain following tibial nailing is indeed a problem that is difficult to treat. The hypothesis of our randomized controlled study was that injury of the infrapatellar nerve contributes to this problem. Anterior knee pain is often multifactorial and infrapatellar nerve injury is one of the causes that has presumably been underestimated. The effect of subcutaneous injection with a local anaesthetic as studied in our trial was never intended as a therapeutic intervention, as we e.g. did not perform a long term follow of the participants. An infrapatellar nerve block with lidocaine can be used as a diagnostic tool to filter those patients who would potentially benefit from denervation of the infrapatellar nerve. Future studies should also focus on avoiding this complication.

**Mandala S Leliveld, Saskia J M Kamphuis, and Michael H J Verhofstad***email:* m.leliveld@erasmusmc.nl
